# Supplementary material for: Human Milk Oligosaccharides in Breast Milk at Two Weeks of Age in Relation to Neurodevelopment in 2-Year-Old Children Born Extremely Preterm: An Explorative Trial
Source: Nutrients. 2025 Feb 27;17(5):832. doi: 10.3390/nu17050832 (PMC11902041; doi:10.3390/nu17050832)
Supplement: Supplementary file 1 [file nutrients-17-00832-s001.zip › nutrients-3481423-supplementary.pdf]

nutrients MDPI

Supplementary Table S1. Drop out analysis

Characteristic	Included, N = 76[1]	Not Included, N = 34[1]	p-value	q-value[2]
Perinatal data				
Gestational age, weeks	25.6 (24.5-26.4)	25.8 (24.4-26.7)	0.6[3]	0.8
Birth weight, g	755 (658-858)	730 (613-829)	0.4[3]	0.5
Birth weight z-score	-0.7 (-1.5--0.2)	-1.1 (-1.5--0.6)	0.084[3]	0.3
Birth length z-score	-0.9 (-1.9--0.2)	-1.5 (-2.3--0.8)	0.032[3]	0.2
Birth head circumference z-score	-0.7 (-1.1--0.3)	-0.8 (-1.4--0.4)	0.2[3]	0.5
Apgar at 5 minutes	6.0 (4.0-8.0)	7.0 (6.0-8.0)	0.090[3]	0.3
Unknown	1	0		
Apgar at 10 minutes	8.0 (7.0-9.0)	8.0 (7.0-9.0)	>0.9[3]	>0.9
Unknown	1	0		
Small for gestational age	14 (18%)	9 (26%)	0.3[4]	0.5
Female sex	32 (42%)	18 (53%)	0.3[4]	0.5
Infants from multiple pregnancy	26 (34%)	15 (44%)	0.3[4]	0.5
Chorioamnionitis	15 (20%)	11 (32%)	0.2[4]	0.4
Caesarean section	45 (59%)	25 (74%)	0.15[4]	0.4
Maternal smoking at inclusion	2 (2.6%)	6 (18%)	0.010[5]	0.2
Prenatal steroids administered	74 (97%)	34 (100%)	>0.9[5]	>0.9
Inclusion site			>0.9[4]	>0.9
Linköping	25 (33%)	11 (32%)		
Stockholm	51 (67%)	23 (68%)		
Neonatal complications				
Sepsis, culture positive	25 (33%)	10 (29%)	0.7[4]	0.9
Intracerebral haemorrhage, grade 3-4	11 (14%)	0 (0%)	0.017[5]	0.10
Periventricular leukomalacia	4 (5.3%)	0 (0%)	0.3[5]	0.9
Necrotizing enterocolitis, grade II-III	7 (9.2%)	2 (5.9%)	0.7[5]	0.9
Days in ventilator, total	15 (4-26)	10 (3-35)	>0.9[3]	>0.9
Bronchopulmonary dysplasia	44 (58%)	22 (65%)	0.5[4]	0.9
Family				
Postgraduate parent, number of			0.8[5]	
0	17 (22%)	9 (26%)		
1	6 (7.9%)	3 (8.8%)		
2	18 (24%)	5 (15%)		
Missing data	35 (46%)	17 (50%)		

[1] Median (25%-75%); n (%)

[2] False discovery rate correction for multiple testing

[3] Wilcoxon rank sum test

[4] Pearson's Chi-squared test

[5] Fisher's exact test

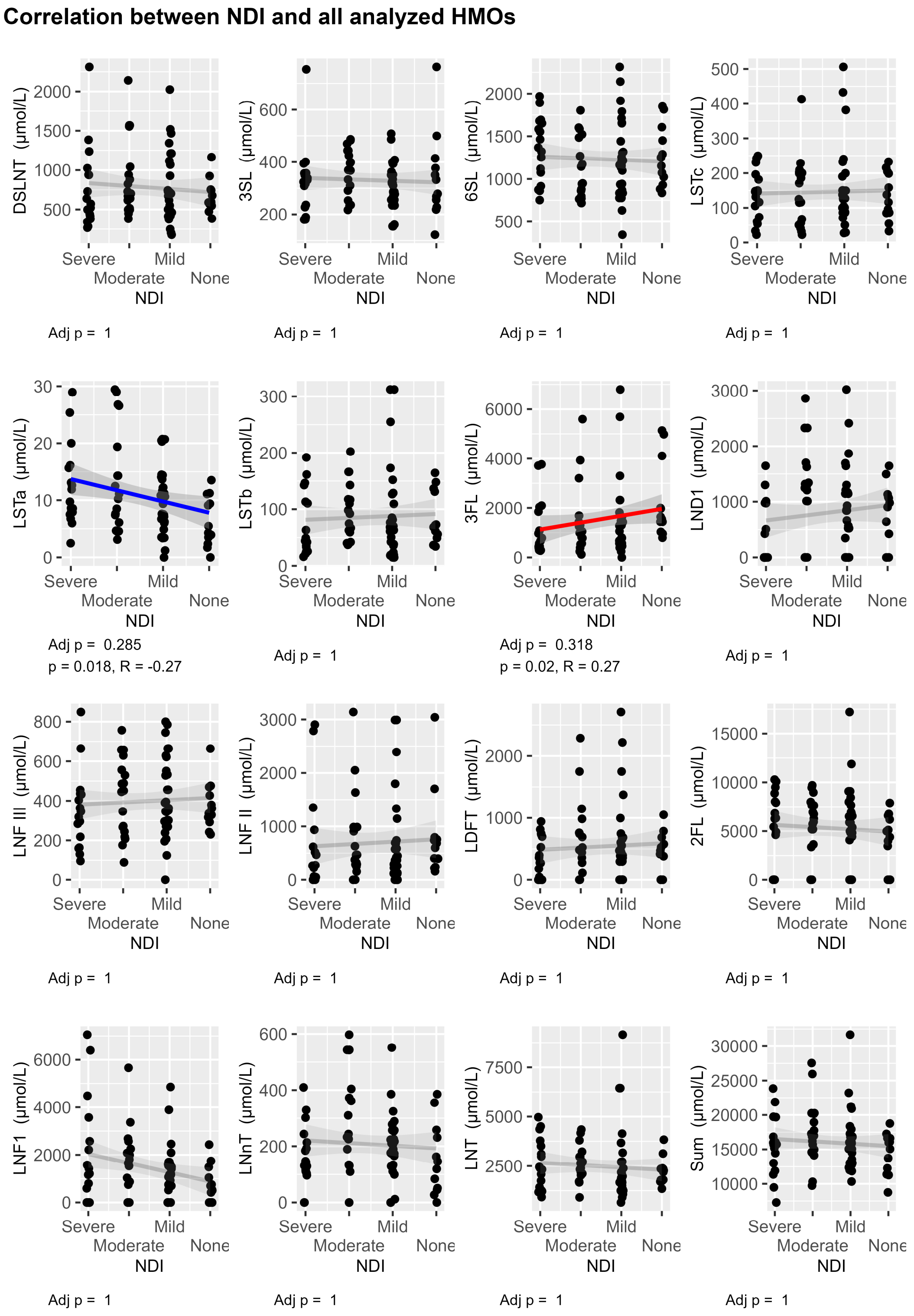

Supplementary Figure S1. The Spearman correlations between HMOs and their total sum versus NDI. Results were not adjusted for multiple comparison in this exploratory analysis.

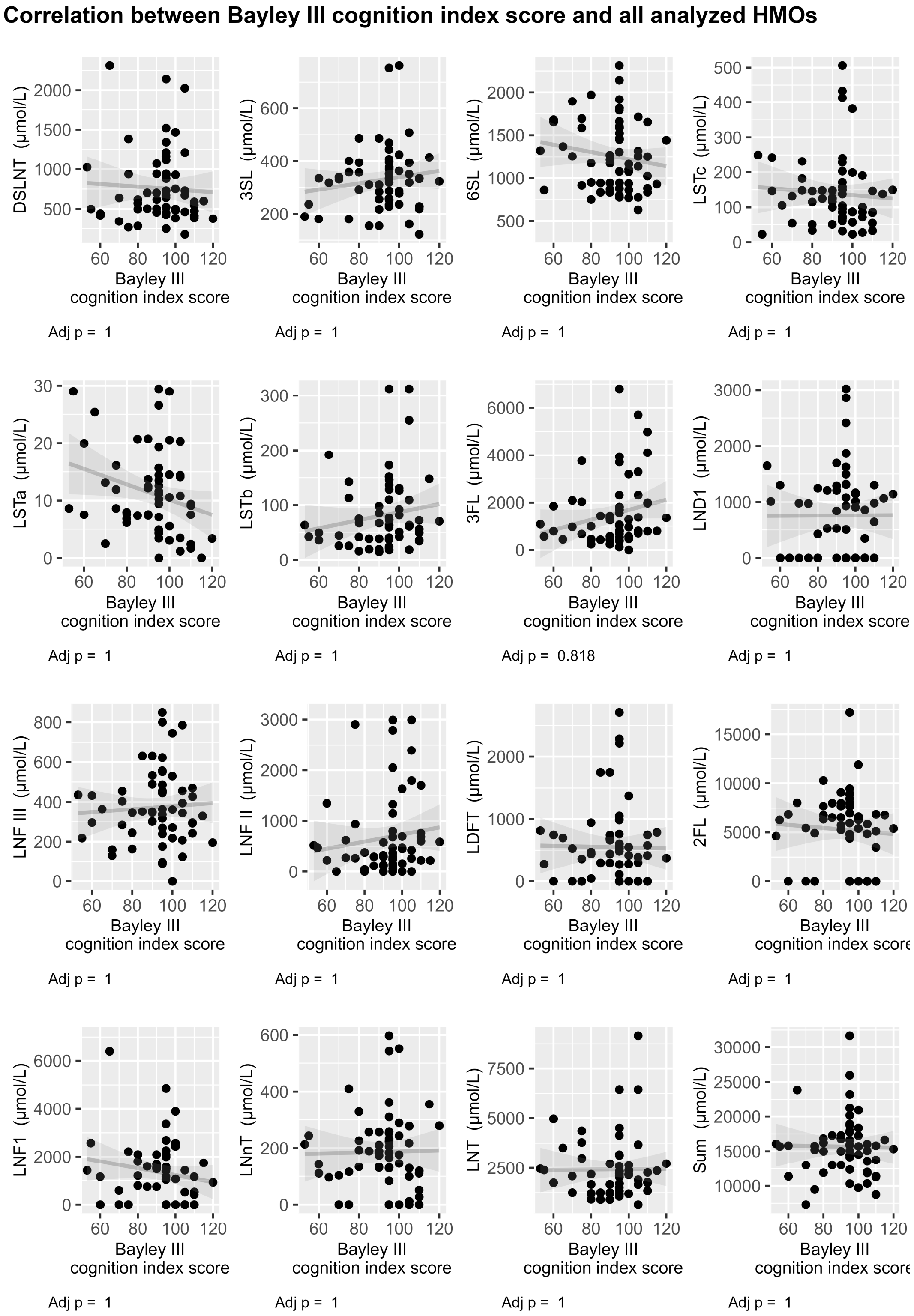

Supplementary Figure S2. The Spearman correlations between HMOs and their total sum versus Bayley III cognition index score. Results were not adjusted for multiple comparison in this exploratory analysis.

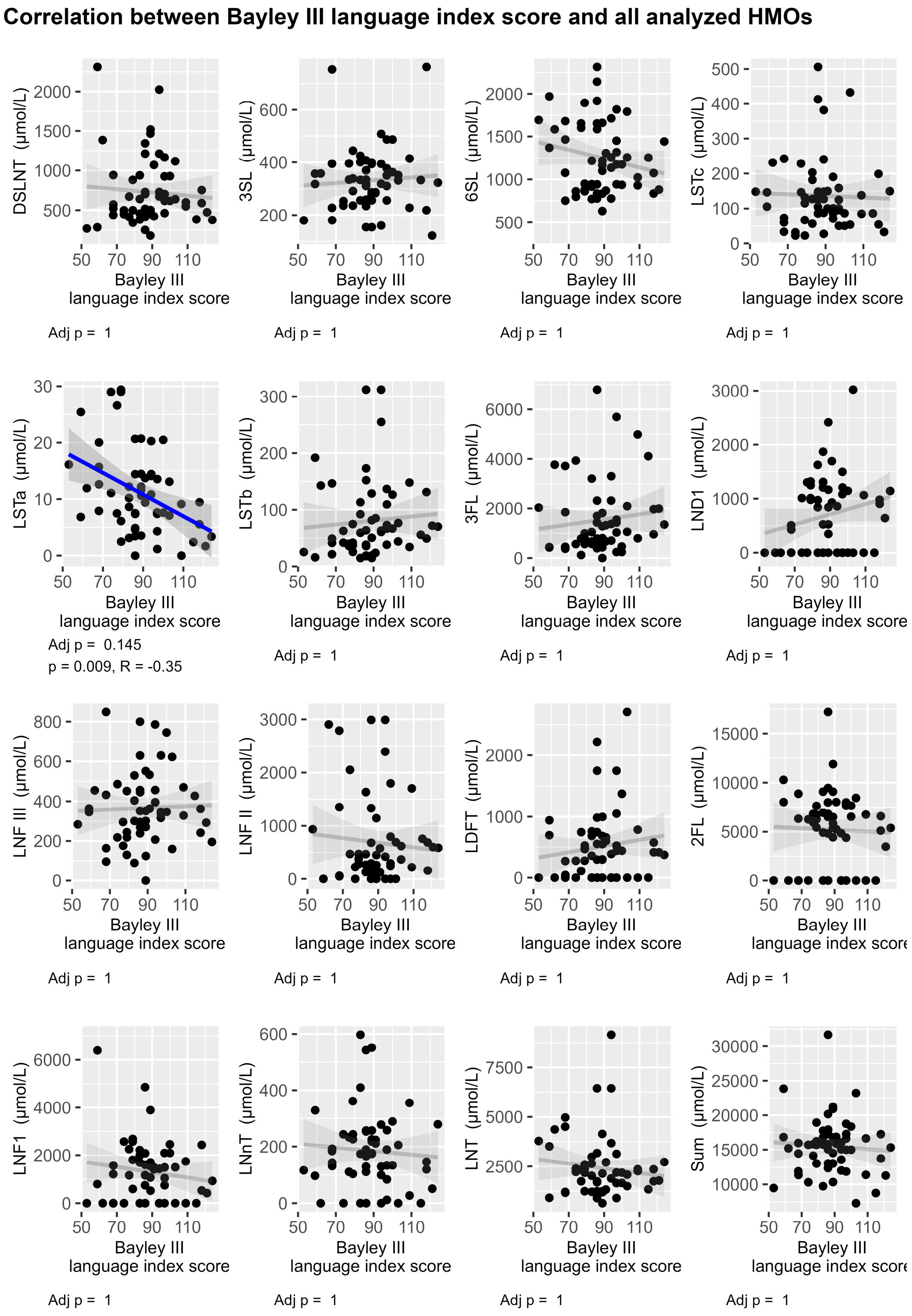

Supplementary Figure S3. The Spearman correlations between HMOs and their total sum versus Bayley III language index score. Results were not adjusted for multiple comparison in this exploratory analysis.

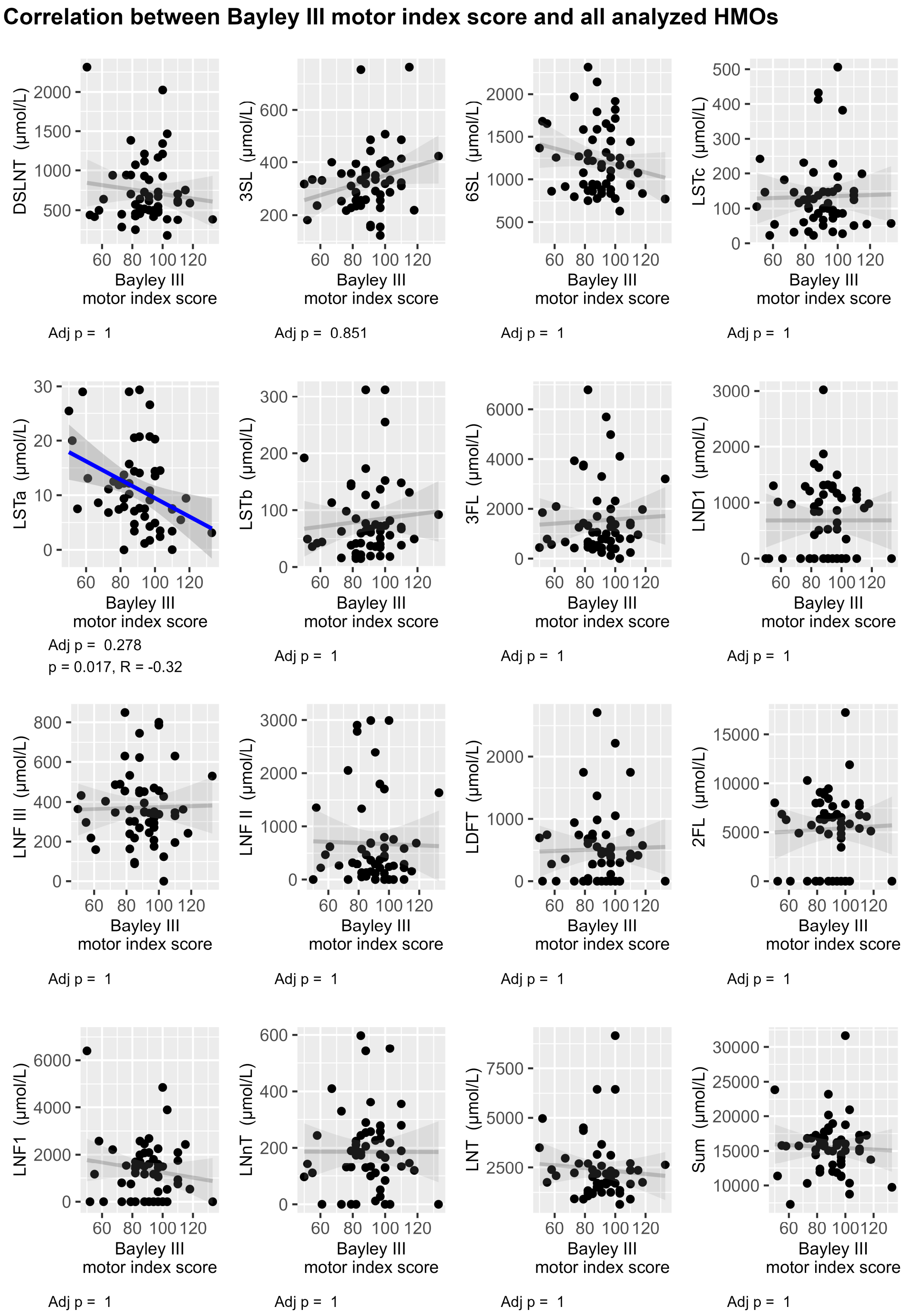

Supplementary Figure S4. The Spearman correlations between HMOs and their total sum versus Bayley III motor index score. Results were not adjusted for multiple comparison in this explora-tory analysis.
